# The Principles of General and Comparative Physiology

**Published:** 1843-04-01

**Authors:** 


THE PRINCIPLES OF GENERAL AND COMPARATIVE PHYSIOLOGY. Intended as an Introduction to the Study of Human Physiology, &c. By *William B. Carpenter*, M.D. Lecturer on Physiology in the Bristol Medical School, &c. Second Edition. London, Churchill, 1841. Royal 8vo. pp. 577.

OUR general opinion of this work has been already made known (Med. Chir. Review, July, 1839). Our notice of the former edition was indeed mostly confined to a brief enumeration of its different sections, a more elaborate survey of its contents having been reserved until it should have reached its present enlarged form. But we are bound to say that no dissatisfaction with the work, even in its original state, prevented our entering into a full examination and analysis of it at the period of its first appearance; for it is throughout a most lucid, comprehensive, and learned exposition of physiological science in all its multifarious ramifications, and one which is adapted to keep its readers fully abreast of all the new discoveries and views elicited by physiologists generally down to the present day. A book like that before us was much needed to fill a glaring hiatus in English literature; and we feel proud to think that, instead of its being a translation from a foreign production, the very plan as well as substance of the work has emanated from the mind of one of our own countrymen. At the same time, the works of foreign writers have been far from disregarded in its progress; and the researches not only of the older but of the most recent physiological inquirers on the Continent—as Müller, Wagner, Ehrenberg, Henle, Valentin, &c.—have been carefully consulted; as well as those of Mayo, Grant, Owen, Lindley, Sharpey, Wilson, Paget, &c. in Great Britain,—by the laborious author. Yet, notwithstanding the magnitude of the task, and the necessity for the extensive research which Dr. Carpenter had imposed on himself, his work has the striking merit of not appearing laborious. The style is easy and clear; and the subjects to which the book refers, which, if treated after a different manner, might have repelled the general reader by their complexity, are, by their mode of exposition, both rendered attractive and readily intelligible to every reflective mind of even the most moderate capacity. We propose in this place to devote a few pages to following the author through the several divisions of his subject, extracting from his book such passages as either most fully and happily display his views, or are otherwise most striking in their character. Before doing this, we may premise that our comments will not, in every instance, be laudatory: but we shall probably terminate our examination (and we here speak both for ourselves and our readers) with the same general impressions as those with which we have begun it.

Commencing his treatise on organised structures with a few words on the distinctions between these and inorganic bodies,—for it has been justly remarked that at whatever point we may begin a history, we shall have to refer to some anterior period,—Dr. Carpenter says—

"In a mineral, every particle possesses a separate individuality, and whatever changes this undergoes in obedience to physical agencies, these changes occur in conformity to laws which apply to it as well separately as in conjunction with others; whilst, in a living being, the action of all parts of the machine are so connected together, that whatever influences one single particle of the organism on which these actions depend, will more or less affect the entire system. Thus we may suppose a mass of gold alloyed with a small quantity of silver, and immersed in nitric acid; this chemical agent will affect every particle of the silver, as completely as if the mass consisted of nothing else, and will leave the gold in its previous condition, having of itself no power of dissolving it. On the other hand, a similar chemical agent applied to an organised structure, will not only destroy the integrity of the part itself, but will produce a disturbance of the general functions proportional to the importance of the organ which has been injured." 7-8.

And in a happy style of phraseology he adds,—

"In the mineral or inorganic world, *change* is the *exception*, and *permanence* is the *rule;* whilst in the animated kingdoms, *change* is constant and universal, and is indeed essential to our idea of life." 9.

How closely allied soever may be the organised kingdoms of Nature (and the Lichens and Sponges seem certainly very nearly connected), a strong line of demarcation is placed between these and the inorganic world. This Dr. Carpenter of course admits. But he singularly enough attributes to the animal and vegetable forms which the more nearly approach the mineral kingdom in some of their characteristics, a greater inherent power of change—rather than a less exalted capacity for it, as would naturally be implied both in their approach towards the condition of mineral bodies, and in their remoteness from the higher forms of animated nature; in which life, with its synonym *change* (see preceding extract), in its highest degree, may be supposed more eminently to reside. Take the following sentences—

"Although, as has been stated, the characteristics of organisation are never so far absent from the living structure as to indicate a transition to the mineral world, it is interesting to remark that, as we descend in the scale of animated creation, we find these peculiarities less striking. And with regard to *form*, it may be observed that this seems least definite among the Sponges and Polypifera (coral-formers) among animals, and among the lowest groups of cellular plants among vegetables; and that there is reason to believe that among these *the same germ may assume a variety of distinct forms*, according to the circumstances under which it is developed, just as the same mineral substance may present itself under a diversity of crystalline shapes." 12.

We cannot pass over this last remarkable admission without observing that the doctrine it conveys, if found to be based on established laws, might prove a powerful engine in the controversy which has long divided philosophers and literati as to the descent of the whole human race from a single stock. If, indeed, it be only understood by the passage printed above in Italics, that many of the organised forms, to which botanists and comparative anatomists had given different specific names, have been since proved to be only different phases of a single species, the deduction is not to be gainsaid. But if, as the hesitating nature of the context would seem to imply, *more* than this be meant, and that *separate species* may be produced from the same germ, we must object to admit the doctrine as any sort of evidence, in the controversy alluded to, from a strong doubt of its validity. Neither does the analogy drawn from the mineral kingdom hold good. Each inorganic substance has in fact its proper qualities and crystalline form impressed on it, and when it loses these, it ceases to be the *same mineral substance*. The notion that the same germ might assume essentially distinct forms is in fact only fit as an argument for the old-fashioned and visionary theorists,—if any such remain,—who have contended for the preposterous idea, that the higher forms of organised nature, including man himself, have proceeded from the progressive development of the lowest germs of organisation into superior forms from the first period of organised creation; an atheistical dream, which nearly all modern physiologists, Dr. Carpenter among the rest, have agreed to explode.

We have been thus critical at the outset for the purpose of warning the reader to examine for himself as he reads; lest he be called on unwittingly to tender his assent to positions which, not bearing the semblance of authenticity, may, if entertained, hereafter embarrass him in his search after truth. Our future observations on particular passages will be generally more brief. But were we to go on step by step through the work, we should meet with many statements, in which we could not wholly coincide, and which would require an equally long examination with the foregoing. Thus when, at p. 14, Dr. Carpenter speaks of the earthy and saline particles in the bony structure of animals as being "evidently subject to no laws but those of physics and chemistry," he decides too hastily upon a question which has by no means been freed from doubt; viz., whether these crystalline particles deposited within the system are to be considered as endued or not with a share of its vitality. It is true that with " the bane," Dr. Carpenter administers " the antidote ;" for in his 46th Section he states, " the osseous tissue retains a higher degree of vitality than the cartilaginous,"—an admission which ought certainly to be considered as overturning his previous decision. But when he asserts (p. 15) that the nervous matter " is constantly being renewed in the living body," we are called on to demand the proof of this, which would rather seem to be contradicted by the absence of lymphatics in the brain and spinal chord—a faith in which absence (whatever may be our own internal impressions on the subject) it is still a matter of philosophical etiquette to hold. The absorbing power of the veins, however, is there.

To Dr. Carpenter's copious survey of the anatomical characteristics of the great divisions of the vegetable and animal kingdoms we cheerfully accord its meed of approbation as a very complete general view of the organised world. But here again observations are often started which call for comment. Thus, the question arises—

" Whether all the fungoid growths on the surface of living plants are really such, or whether they may be regarded as degenerations of the tissue upon which they are found. Unger, a German botanist, has argued with considerable ingenuity that the appearances termed blight, mildew, smut, &c. are to be considered as the *exanthemata* (eruptive fevers) of vegetables, being essentially diseases of the stomata. * * * It remains a question, which we are yet scarcely in a condition to answer without reserve, either in one way or the other, whether plants of a high degree of organization are capable of producing, by diseased action, from various parts of their tissues, beings which present the characters of inferior orders. * * * We shall hereafter see that the function of reproduction may be considered as only a peculiar modification of that of nutrition; and if its *regular* performance leads to the evolution of germs, which, when developed, resemble the parent, it is not irrational to suppose that it may be so far perverted, as to give origin to beings of simpler organisation." 73.

Now the enunciation of this opinion is a retrograde march to the proposition which we have already combated, of essentially different organised forms being generated from the germ of a single organised species. We are naturally bound to exclaim,

Nec Deus intersit, nisi dignus vindice nodus
Inciderit— Hor.

Why call in a new power, if the effect be referrible to those we know of as already existing? It is surely more consonant with a philosophic spirit first to examine narrowly if laws and arrangements, of the truth of which we are already assured, be capable of producing the phenomena which we witness. How can we doubt, when Fries has counted in a single fungus (*reticularia maxima*) upwards of 10,000,000 sporules, so subtle, as Dr. Carpenter himself observes, that they are " scarcely visible to the naked eye, and often resemble thin smoke, so light that it is difficult to conceive a place from which they can be excluded, and dispersed in so many ways by the attraction of the sun, by insects, wind, elasticity, &c."—why need we doubt that these and the germs of other species, at their time of maturity, float in myriads through the atmosphere ready to develop their parent forms in whatever soil (such as the diseased secretions of other plants) may be best adapted for fostering their growth?—an hypothesis unequivocally more conformable with probability than any other which has been offered to account for the development of parasitic plants. That germs are carried and plants are developed in this way, is proved by the following curious fact.

"Individuals of a species of *Polistes* (the *wasp* of the West Indians) are often seen flying about with plants of their own length projecting from some part of their surface; the germs of which have been introduced, probably through the breathing pores at their sides, and have taken root in their substance, so as to produce a luxuriant vegetation." 73.

We should hardly have expected, *à priori*, to find *Zoophytes* so high in the scale of creation.

We shall close our notice of what is called the introductory portion of the work by the following passages, which place before the reader pleasing, if fanciful, analogies between the great divisions of the animal and vegetable kingdoms.

"The correspondence of ARTICULATA with ENDOGENS, as well as that of VERTEBRATA with EXOGENS, was long ago pointed out by Desfontaines, who first discovered this primary natural division of the Phanerogamia founded on the structure of their stems. Thus both of the groups just mentioned have their hardest portions, or organs of support, placed externally; in both, the additions to their tissue are formed from within; in both, also, there is usually a distinct division into segments, each of which, in plants, as in the lower Annulose animals, contains the organs essential to its vitality; and in Endogens, as in Insects, are the *tracheæ* (respiratory tubes) distributed through the whole system. The hard external parietes of these classes undergo little or no change in diameter when once fully formed; but Endogenous plants have not that power of occasionally throwing them off, which is possessed by most of the Articulated animals.

"In like manner, EXOGENS may be considered analogous to VERTEBRATA, in the internal situations of their hard parts, the formation of new tissue from without, the less distinct division into segments, and the confinement of the internal respiratory apparatus to a particular situation in the fabric." 137.

Book 1, which treats of General Physiology, opens with a chapter on the Nature and Causes of Vital Actions, comprising a disquisition on the subject of the vital principle, which, as it has been brought under review on a former occasion (Med. Chir. Review, July 1839), we shall for the present pass over in silence; though it will be alluded to once more before the termination of the present article. Heat, light, and electricity, as vital stimuli affecting the organism; and some of the effects of food and air in modifying the growth and development of the body, are the subjects next entered upon. Some extracts from these sections will be found interesting: for instance, the description of the following curious effect of food in modifying the development of the frame in one of the lower classes of animals.

"In every hive of bees, the majority of individuals consists of *neuters*, which have the organs of the female sex undeveloped, and are incapable of reproduction; that function being restricted to the *queen*, who is the only perfect female in the community. If by any accident the queen be destroyed, or if she be purposely removed for the sake of experiment, the bees choose two or three from among the neuter eggs that have been deposited in their appropriate cells, which they have the power of converting into queens. The first operation is to change the cells in which they lie into *royal* cells, which differ from them considerably in form, and are of much larger dimensions; and when the eggs are hatched, the maggot is supplied with food of a very different nature from the farina or bee-bread which has been stored up for the nourishment of the workers, being of a jelly-like consistence and a pungent stimulating character. After the usual transformations the grub becomes a perfect queen, differing from the neuter bee into which it would otherwise have changed, not only in the development of the reproductive system, but in the general form of the body, the proportionate length of the wings, the shape of the tongue, jaws, and sting, the absence of the hollows on the thighs in which the pollen is carried, and the loss of the power of secreting wax." 169.

"The following curious fact may be introduced here, as having an interesting analogy with that just now stated regarding the bee. It is mentioned by Mr. Knight that cucumber and melon plants will afford all male or staminiferous flowers if vegetation be accelerated by heat; and all female or pistilliferous, from the same points, if its progress be retarded by cold." 173.

The influence of light in favouring animal development is strikingly pointed out in the following passage.

"The influence of light on Animal development has been proved in the most striking manner by the experiments of Dr. Edwards. He has shown that if tadpoles be nourished with proper food, and are exposed to the constantly-renewed contact of water (so that their branchial respiration may be maintained) but are entirely deprived of light, their growth continues, but their metamorphosis into the condition of air-breathing animals is arrested, and they remain in the form of large tadpoles. Dr. E. also observes that persons who live in caves or cellars, or in very dark and narrow streets, are apt to produce deformed children; and that men who work in mines are liable to disease and deformity, beyond what the simple closeness of the atmosphere would be likely to produce. It has been recently stated, on the authority of Sir A. Wylie, that the cases of disease on the dark side of an extensive barrack at St. Petersburgh, have been uniformly, for many years, in the proportion of three to one, to those on the side exposed to strong light. On the contrary, the more the body is exposed to the influence of light, the more freedom do we find, *cæteris paribus,* from irregular action or conformation. Humboldt has remarked that, among several nations of South America who wear very little clothing, he never saw a single individual with a natural deformity; and Linnæus, in his account of his tour through Lapland, enumerates constant exposure to solar light as one of the causes, which render a Summer journey through high northern latitudes so peculiarly healthful and invigorating." 180.

This passage affords a strong testimony to the practical benefits which scientific enquiries are capable of producing to mankind, if only a due attention be paid to the results which they bring into view.

In the chapter on the laws of Organic Development, the great law of *unity of composition,* by which the various forms of animated nature (as is especially seen in the vertebrated classes) are modifications of an uniform type, is broadly laid down.

"Thus it would be easy to show that the skeleton of a fish is formed of the same parts as that of a bird or quadruped; though the form of each individual bone may be totally dissimilar in the two cases. Again, we have seen that the lung of the air-breathing Vertebrata exists in a rudimentary condition in fishes, whilst the rudimemts of a branchial apparatus are found in the embryos of the higher classes."

"The skeleton essentially consists of a jointed column of bones enclosing the nervous system, with which certain appendages are connected for various purposes. The different parts of this column appear in the highest classes, very dissimilar to one another,—thus, the bones of the skull have no apparent resemblance to the vertebræ of the back; and these seem but very imperfectly represented in the joints of the tail. Further examination, however, will show that the skull is but an expansion of the three highest vertebrata, modified to afford space for the development of the contained brain, and of the organs of sense; and, however strange such a statement may appear to those who are only acquainted with the cranium of man, the fact is evident where the brain is little developed, as in the lower reptiles and fishes." 191.

But in tracing up the history of the functions of the animal frame from its lowest to its highest system of development,—a task which he has executed with signal ability—Dr. Carpenter still gives in occasionally to opinions so unfounded and startling as to call for a decided protest. Thus he asserts,—

"There is no good reason to believe that 'nervous agency' is essential to the processes of nutrition and secretion in animals, any more than to the corresponding processes in plants. * * * Observation shows us that these processes are performed in the most complex and elaborate manner by vegetables, in which all the attempts that have been made to prove the existence of a nervous system have signally failed, (these attempts seeming to have been only excited by an indisposition to admit the possibility of any vital actions being independent of nervous influence); that the lowest animals appear equally destitute of a nervous apparatus destined to influence them." 209.

By the far greater number of physiologists, the performance of these functions has been attributed to the agency of the sympathetic nervous system—a system, the presence of which, together with that of a digestive cavity, we would propose as the best definition of an animal, as contradistinguished from a vegetable organism. For though it may not yet have been demonstrated by microscopical observation, all analogy still warrants us in presuming on the presence of a nervous system in even the lowest animal forms; and this system will assuredly be the ganglionic, or that to which the nutritive and other involuntary functions are subservient—the voluntary nervous system (which in the Vertebrata becomes the cerebro-spinal) not being superadded till we ascend higher in the scale of beings. Nay, it has even been advanced, that no animal tissue is formed except coetaneously with the nervous matter supplied to it. And the circumstance of plants performing their various functions without the agency of a nervous system, is an instance altogether futile for the argument which the author wishes it to subserve; being only one of the many proofs that the Divine Author of Nature can effect analogous ends by widely dissimilar means. For examples of this we need not step beyond the animal kingdom itself. We see the function of trituration of the food, which in one vertebrated class is performed in the mouth, to be in another class performed in the stomach; and that motion through the air which in the bird is effected by a true wing, is effected in the insect by a true respiratory organ.

In that portion of the work in which the functions of digestion and absorption are traced upwards in the animal kingdom, we are called on to notice the measure of success which has attended the experiments of some German physiologists in forming an *artificial* gastric juice. This has been formed, by Müller and Schwann,

"Of a mixture of dilute acetic or muriatic acid with mucus of the stomach; the simplest way of manufacturing it being, to macerate a portion of mucous membrane in the acid. Neither acids nor mucus will act alone; but the correspondence of their united effect, so far as it goes, with that of the gastric juice, can leave no doubt that the operation of the latter is of a chemical nature; it has been recently shewn, by Purkinje and Pappenheim, that the influence of galvanism enables saliva or mucus alone to dissolve albumen, by decomposing the chloride of sodium which these fluids contain, and thus liberating muriatic acid. There is no difficulty, therefore, in accounting for the presence of muriatic acid in the stomach." 236.

And in the same section, the remarks on the *natural* secretion of the gastric juice might not be without their moral usefulness, if read and attended to. We particularly commend them to "the English epicures."

"It appears from the experiments and observations of Dr. Beaumont, that the secretion of gastric juice does not continue in proportion to the quantity of food taken into the stomach, although at first excited by its contact, but is regulated by the wants of the system; so that, when sufficient nutriment has been provided for absorption, no further active process of digestion goes on, although the *will*, inattentive to the dictates of Nature, continues to transmit to the stomach more food than is dissolved at the time. Vegetables cease to absorb when the structure is replete with food, and there is no continued demand for it. The more we pursue our remarks into the actions of plants and of the lower tribe of animals, the more we are struck with the beauty of the adaptations, by which the influence of a capricious will, which would often be to the injury of the system, is rendered unnecessary." 238.

We are next drawn to notice the following view of alimentation.

"It is curious to observe, in the progress of the food along the alimentary canal of higher animals, the gradual removal of it from connexion with the functions of (so-called) *animal* life. To procure it, in the first instance, is one important office of these functions; and the highest exercise of the locomotive, sensorial, and intellectual powers is often required for this purpose. Its introduction into the mouth is an act of pure volition in man; whilst the masticatory movement to which it is there subjected, may be regarded as having been originally voluntary, but as afterwards so completely habitual as to be scarcely dependent on the will, although not removed from its control." 237.

An able summary of the progressive development of the digestive tube closes the chapter on digestion.

"Its simplest evolution may be seen in the gemmules of the Sponges, which at first are permeated by no canals; but, as they fix themselves, depressions are seen on their surface, which gradually deepen into tubes, and these ramify and unite to form the system of passages peculiar to these beings. In the Radiata, in general, it appears to be formed simply by the inner layer of the germinal membrane, which spreads itself over the yolk, and forms a bag, at first closed, but afterwards perforated by an orifice which constitutes the mouth. Its most complex forms may be traced from an equally simple commencement; for in the embryos of the Vertebrata the intestinal canal first exists, as a straight tube, formed by a fold of the germinal membrane; thus evidently corresponding with its condition in the lower Annulose tribes. The two ends of this tube are at first closed, the middle portion opening into the yolk-bag, which contains its store of temporary nutriment. In the human fœtus, the oral opening is formed at the sixth week, the opposite one a week later; sometimes the latter remains closed even until birth. The stomach is first distinguished, by a projection of the tube towards the left side, about the ninth week; but the separation of the small and large intestines is of much later occurrence. The folds of the mucous membrane, which are confined to higher animals, do not appear until a late period of gestation." 250.

From what has already been advanced, our readers will readily have gathered that we think higher of Dr. Carpenter when he confines himself to description than when he wanders in the mazes of conjecture. Such is the fact. We think better of his judgment, than of his fancy. He is an excellent compiler: he seizes the facts which are laid before him—condenses, prunes, elucidates—and describes the thoughts or the observations of others, in concise terms and in good language. And, this being the case, our future extracts, which must be few, will be principally confined to examples in which he has recorded plain facts in the most happy style.

The book improves as it proceeds; but want of space compels us to pass over the chapters on Circulation, Absorption, Nutrition, and Respiration, with little comment, The last of these chapters comprises Dr. Carpenter's own view of the respiratory process. The detail of this view is too lengthy to insert here, and it deserves to be studied in an unmutilated form. We may remark, however, that the author includes under the phenomena of respiration, the absorption of nitrogen by the lungs, (p. 368.) In this, his opinion is directly opposed to that of Liebig, who plainly asserts that " no nitrogen is absorbed from the atmosphere in the vital (respiratory) process."—(Liebig and Gregory, &c. p. 43 ; in which work the subject is discussed at length.)

On the subject of exhalation from the surface in animals the following statement is made, which may furnish a good starting-point for more extended inquiries in this department of physiology.

" From the experiments of Lavoisier and Seguin it appears, that the maximum quantity of fluid exhaled from the cutaneous and pulmonary surfaces in man is 5lbs. per day, the minimum being 1¾lb.; and that the mean quantity exhaled per minute is 18 grs., of which 11 pass off by the skin, and 7 by the lungs. There is much difficulty in attaining correct information on this subject, however, owing to our ignorance of the amount absorbed from the atmosphere; and that, under favorable circumstances, the quantity of fluid exhaled from the skin may be much greater in a short time than these results would lead us to believe, appears from the late observations of Dr. S. Smith. These were made upon labourers at the Phœnix Gas Works, who are employed twice a day in drawing and charging the retorts and in making up the fires; which usually occupies about an hour; the labour is performed in the open air, but is attended with much exposure to heat. On a foggy and calm day at the end of November, when the temperature of the external air was 39°, and the men continued at their work for an hour and a quarter, the greatest loss observed was 2lbs. 15oz.; and the average of eight men was 2lbs. 1oz. On a bright clear day in the middle of the same month, when the temperature of the air was 60° without much wind, the greatest loss was 4lb. 3oz.; and the average was 3lb. 6oz. And on a very bright and clear day in June, when the temperature of the external air was 60° without much wind, the greatest loss (occurring in a man who had worked in a very hot place) was 5lbs. 2oz.; and the average during the hour was 2lb. 8oz." 385.

These observations are succeeded by chapters on Secretion, the Evolution of Heat, Light, Electricity, &c. in Organic Beings. In the section on the evolution of heat, the following remarks appear, derived from a paper in the Philosophical Transactions by Mr. Newport.

" In the *Larva* condition the temperature of the animal corresponds much more closely with that of the atmosphere than in the perfect state; thus, the larva of the higher species of *Hymenoptera* (humble-bees, &c.), is usually from 2° to 4° above the surrounding medium, whilst the perfect insect has a range of from 3° to 10°, or even more; and the caterpillar of the *Lepidoptera*, (butterfly tribe,) is seldom more than from ½° to 2° warmer than the atmosphere, (the amount varying in close relation with the activity of the individual;) whilst the perfect insect is, when much excited, 5° or 9° above it." 421.

The writer of the present article is glad to perceive that extensive use has been made, not only in the above place, but throughout many appropriate parts of the work, of the accurate and valuable researches of our countryman Newport, a personal knowledge of whom enables him to bear testimony to the singular zeal and industry of the latter in entomological enquiry. This is at the same time a fitting place to accord due praise to the indefatigable zeal of Dr. Carpenter, who has spared no pains in the search after all authentic information upon physiological science, wherever it was to be found.

On electrical development in certain animals, many interesting considerations are brought together. In tracing the nerves of the electrical apparatus in the principal electrical fishes, Dr. Carpenter remarks,—

" In all these instances, the electrical organs are supplied with nerves of very great size, larger than any others in the same animals, and larger than any nerve in other animals of like bulk. They all arise in the Torpedo from the cranial portion of the cerebro-spinal axis; of these, the two first issue from the cranium in close proximity with the 5th pair, and have been regarded as belonging to it, although their real origin is different; and, from the third electrical nerve to the stomach, after sending its principal portion to the electrical organs, it would seem analogous to the 8th pair. The electrical nerves in the Gymnotus are believed to arise from the spinal marrow alone; and those of the Silurus are partly intercostals, and partly belong to the 5th pair." 438.

Now the circumstance that the electrical nerves in the Torpedo should be analogous to the 8th pair in the higher Vertebrata is one of a highly striking nature. Of all nerves in the human subject, the 8th pair (the par vagum) is that which, with the organs to which it is distributed, appears to exhibit the most intimate sympathising connexion with cerebral impressions. The influences of fear and anger (which are probably the chief exciting causes of the instinctive electric discharges of the Torpedo), of hope, affection, and indeed of all passions, whether of an exciting or depressing kind, are inevitably manifested more or less on the heart, lungs, stomach, larynx, &c., and which derive their nervous influence partly through the branches of the par vagum; and these manifestations are often attended with considerable alterations in the secretions of these organs or a great exaggeration of their vitality. Nay, the analogy is even further carried out by pathology. For in hydrophobia—a disease in which the nervous energy is, in paroxysms, exalted to the highest pitch, and the secretions of parts to which the 8th pair is supplied, are exasperated into a poisonous quality,—the chief pathological lesion discovered after death has been *said* to be an inflammatory state of the trunk of the 8th pair where it issues from the skull.

We have no hesitation in stating, that the chapter on Reproduction is the best part of the whole work. But we can spare room only for the following comprehensive extract.

"All organised beings, without exception, take their origin in a *cell* or vesicle; and there is nothing in the appearance or character of the embryonic cell of the animal, to distinguish it from that of the plant, or from the permanent cells of the simplest vegetable fabrics. This form of structure, therefore, is at the same time the simplest and most universal in the organised world; and it embodies the whole idea of organisation. It consists of a solid and fluid part—a containing and contained portion,—acting and re-acting upon each other. From this *most general* condition, therefore, of an organised body, which is the first that manifests itself in the production of a new structure, a more special form soon evolves itself; for changes speedily take place in the character of the embryonic mass, which enables the observer to determine the *kingdom* to which it belongs. At first, however, there is nothing in its aspect which determines its more special type or *sub-kingdom,*—Radiated, Annulose, Molluscous, or Vertebrated; but this is next evolved. The characters of the particular *class* then manifest themselves; the embryo of the Mammal, for example, is known from that of the Bird, by the difference in the changes which take place in reference to the yolk-bag, before the particular *order, family, genus,* and *species* to which it belongs, can be determined from its structure. These are progressively manifested,—the original law of the elaboration of a special type out of one more general being closely applicable here. The characters of the *sex* make their appearance at a later period; but it is not until *birth* that those of the individual properly display themselves." 499-500.

*Deus dabit his quoque finem.* We are rapidly reaching the utmost extent of our limits. But the planting of our god Terminus must be for a few instants arrested, while we cite, in Dr. Carpenter's own words, the expression of an idea, on the hereditary transmission of acquired powers, in which we fully and warmly concur.

"No one, who has had sufficient opportunities of observation, can doubt that the intellectual faculties, which have been developed by cultivation, are generally transmitted to the offspring in an improved state; so that the descendant of a line of educated ancestors will probably have a much higher capacity for instruction than the child that springs from an illiterate race." 512.

How wide a field for discussion and for action does this consideration present! How gravely do the spread of education and the *science* of intermarriage address themselves to the attention of the philanthropist and the legislator! The fruits of care and culture are not confined to the well-being of a single individual: they bear within them the blessings of increase; and multiply tenfold with each succeeding generation.

In the notice of the first edition of this work in the Medico-Chirurgical Review, all special observation was confined to an exposition of Dr. Carpenter's view of the Nature of the Vital Principle, chiefly for the purpose of affording a comparison between it and the theories of other authors upon the same subject. If some of these theories are absurd or mischievous, the same complaint is not applicable to that of Dr. Carpenter. This may exhibit little that is novel; but neither has it any thing presumptuous:—in it is no attempt "to fathom the unfathomable," which is so much the characteristic of conceited ignorance, or German transcendentalism. In all its broad lineaments, Dr. Carpenter's portrait of vitality agrees with that of Liebig, and as we have already, in another place, travelled side by side with Dr. Carpenter in his journey of discovery after it, we will not go over the same ground again, but refer to what Liebig has to say on the subject;—chiefly for the purpose of filling up our Bombastes' army, if not "awkward squad" of theories respecting the "vital principle." Liebig then,—who judges of the cause itself from its effects; I. As a force of attraction; II. As a means of resistance; and III. As a cause of motion in the material frame—considers Vitality as "a peculiar property, which is possessed by certain material bodies, and becomes sensible when their elementary particles are combined in a certain arrangement or form;" but which remains occult in matter until its elements have assumed such an arrangement or form. The origin of the vital force he attributes to a change of matter: but while he admits that "the laws of vitality, and of all that disturbs, promotes, or alters it, may be discovered," he still frankly acknowledges that "we shall never learn what life really is —of what it essentially consists."* Drawn once more to the contemplation of this mysterious secret as the proper termination of his theme, Dr. Carpenter winds up his labours with the following truly eloquent peroration.

"It has been one object of the foregoing pages to show that *vital* properties are as essentially connected with certain forms of matter, as are those usually denominated *physical* with matter under its more common aspects. One more question yet remains. Is it possible that the physical and vital properties of matter, which are at present our ultimate facts or axioms, may be included within a more general expression common to both? On this subject we can only speculate; but the probability appears decidedly in the affirmative. It has already been remarked, that the rapid progress of generalisation in physical sciences renders it probable that, ere long, a simple formula shall comprehend all the phenomena of the inorganic world; and it is not, perhaps, too much to hope for a corresponding simplification in the laws of the organised creation, although its progress is necessarily retarded by the many obstacles, which the nature of the subject presents to the philosophic enquirer. Every step which we take in the progress of generalisation, increases our admiration of the beauty of the adaptation, and the harmony of the action, of the laws we discover; and it is in this beauty and harmony that the contemplative mind delights to recognise the wisdom and beneficence of the Divine Author of the Universe. This, in fact, is one of the highest results to which the exercise of our intellectual faculties should lead; and we cannot but believe, that the Creator, in endowing us with these faculties, intended that they should conduct us nearer to the conception of His Infinite mind. But, at the same time, the vastness of the prospect thus disclosed, can scarcely fail to impress us with the most humbling consciousness of our own insignificance.

---

* Extracted from Mr. Ancell's Exposition of Liebig's Views, in the Lancet, 1842-3, Vol. ii, 232-235.

"If then we can conceive that the same Almighty *fiat* which created matter out of nothing, impressed upon it one simple law, which should regulate the association of its masses into systems of almost illimitable extent, controlling their movements, fixing the times of the commencement and cessation of each world, and balancing against each other the perturbing influences to which its own actions give rise,—should be the cause, not only of the general uniformity, but of the particular variety of their conditions, governing the changes in the form and structure of each individual globe, protracted through an existence of countless centuries, and adjusting the alternation of 'seasons and times, and months and years,'—should people all these worlds with living beings of endless diversity of nature, providing for their support, their happiness, their mutual reliance, ordaining their constant decay and succession, not merely as individuals but as races, and adapting them in every minute particular to the conditions of their dwelling,—and should harmonise and blend together all the innumerable multitude of these actions, making their perturbations sources of new powers;—when our knowledge is sufficiently advanced to comprehend these things, then shall we be led to a far higher and nobler conception of the Divine Mind than we have at present the means of forming. But, even then, how infinitely short of the reality will be any view that our limited comprehension can attain, seeing, as we ever must in this life, 'as through a glass, darkly;'—how much will remain to be revealed to us in that glorious future, when the light of truth shall burst upon us in unclouded lustre, but when our mortal vision shall be purified and strengthened so as to sustain its dazzling brilliancy!" 563.

We now close this volume, and look back upon it, *as a whole,* with the same sentiments as those we entertained of it at the commencement. If we have seemed querulous or occasionally hypercritical, it has been because it is natural to wish that which we admire to be free from blemishes:—and these invariably stand out more prominently, the more attractive the field that they disfigure. Unqualified praise or censure belongs to comparatively few books. But taking this one "with all its faults" we can honestly say that we have met with few in which more truth is mixed with less error.

